# Functional Analysis of Odorant-Binding Proteins 12 and 17 from Wheat Blossom Midge *Sitodiplosis mosellana* Géhin (Diptera: Cecidomyiidae)

**DOI:** 10.3390/insects11120891

**Published:** 2020-12-17

**Authors:** Weining Cheng, Yudong Zhang, Jinlin Yu, Wei Liu, Keyan Zhu-Salzman

**Affiliations:** 1 Key Laboratory of Plant Protection Resources & Pest Management of the Ministry of Education, College of Plant Protection, Northwest A&F University, Yangling 712100, China; zydskrr@gmail.com (Y.Z.); yujinlin91@nwafu.edu.cn (J.Y.); cherryapple788@gmail.com (W.L.); 2 Department of Entomology, Texas A&M University, College Station, TX 77843, USA

**Keywords:** *Sitodiplosis mosellana*, odorant-binding protein, wheat volatiles, fluorescence binding assay, behavioral assay

## Abstract

**Simple Summary:**

*Sitodiplosis mosellana* is one of the most destructive pests of wheat. Adults rely highly on wheat spike volatiles to search and locate oviposition sites. Insect odorant-binding proteins (OBPs) are important in binding and transporting host plant volatiles to the olfactory receptors. Therefore, OBP-based behavioral interference is believed to be a novel and effective pest management strategy. The objectives of this study were to clone two *S. mosellana* female antenna-enriched *OBP* genes (*SmosOBP12* and *SmosOBP17)*, determine the functions of the encoded SmosOBP proteins in binding wheat volatiles, and investigate behavioral responses of female *S. mosellana* to odorant molecules. Results indicated that SmosOBP12 had a broader ligand-binding spectrum than SmosOBP17 to wheat volatiles. Female *S. mosellana* showed intensive response to 3-hexanol, 1-octen-3-ol, D-panthenol, 3-carene, (*Z*)-3-hexenylacetate, hexyl acetate, methyl salicylate, heptyl acetate, ethyl heptanoate, α-farnesene, and ocimene. Notably, all these compounds except α-farnesene exhibited strong affinity to SmosOBP12. In conclusion, SmosOBP12 may play more crucial roles than SmosOBP17 in perception and transportation of biologically active host volatiles. This information has enhanced our molecular understanding of the *S. mosellana* olfaction, which could also serve as an important reference for developing attractants or repellents to control this pest.

**Abstract:**

The wheat blossom midge *Sitodiplosis mosellana*, one of the most disastrous wheat pests, depends highly on olfactory cues to track suitable plants. To better understand the olfactory recognition mechanisms involved in host selection, in the present study we cloned two *S. mosellana* adult antenna-specific odorant binding protein (OBP) genes, *SmosOBP12* and *SmosOBP17*, and evaluated bacterially expressed recombinant proteins for their selectivity and sensitivity for host wheat volatiles using the fluorescence-based ligand binding assay. The results showed that both SmosOBPs effectively bound alcohol, ester, ketone, and terpenoid compounds. Particularly, SmosOBP12 had significantly higher affinities (K_i_ < 10.5 μM) than SmosOBP17 (K_i_2 > 0.1 μM) to 3-hexanol, 1-octen-3-ol, D-panthenol, 3-carene, (*Z*)-3-hexenylacetate, hexyl acetate, methyl salicylate, heptyl acetate, and ethyl heptanoate. Consistently, *S. mosellana* females were attracted to all these chemicals in a behavioral assay using Y-tube olfactometer. SmosOBP12 also bound aldehyde, but neither bound alkanes. Notably, SmosOBP12 exhibited strong affinity to ocimene (K_i_ = 8.2 μM) that repelled *S. mosellana*. SmosOBP17, however, was insensitive to this compound. Taken together, our results indicate that SmosOBP12 may play a greater role than SmosOBP17 in perceiving these biologically active plant volatiles.

## 1. Introduction

Important insect behaviors such as host plant selection, mate finding, and oviposition site searching are mediated by volatile chemical signals released from plants and conspecific partners [1,2,3,4]. Insects rely on their highly sensitive and specific olfactory systems to detect and discriminate these semiochemicals [5,6,7]. As the initial step of odorant reception, small water-soluble carrier proteins, namely the odorant-binding proteins (OBPs), selectively bind and transport external odorant molecules through the aqueous sensillar lymph to specific odorant receptors (ORs) on the dendrite membrane of olfactory neurons, activating the signal transduction pathway [8,9]. OBPs thus could be potential targets in the effort of interrupting chemical communications within species, and between insect pests and their host plants. Such an indirect insecticidal approach could play a crucial role in integrated pest management, broadening the arsenal of available tools for pest monitoring and control [10,11,12].

The first insect OBP was identified in the antennae of male *Antheraea polyphemus* using a radiolabeled pheromone [13]. With the development of molecular and high-throughput sequencing techniques, a large number of OBPs have been identified from insects belonging to at least eight different orders [14,15,16,17,18,19,20]. They all possess six conserved cysteine residues which form three disulfide bridges. These OBPs are generally classified into two subfamilies, namely general OBPs (GOBPs) and pheromone-binding proteins (PBPs) based on amino acid sequence homology and ligand specificity [21]. It is suggested that PBPs are male antenna-specific and respond mainly to pheromone components [22,23,24,25], whereas GOBPs are mainly expressed in antennae of both sexes and interacted with host plant volatiles [26,27,28]. Multiple OBPs with distinct functions can generally be found within a single insect species [29,30,31]. For instance, GmolOBP10 of the oriental fruit moth *Grapholita molesta* has very strong affinities to hexanol and dodecanol while GmolOBP4 does not bind to these two compounds at all. Instead, the best ligands for GmolOBP4 are hexanal and pear ester [32]. MmedOBP2 from parasitic wasp *Microplitis mediator* mainly binds aromatics, but MmedOBP6 primarily interacts with terpenoids, and MmedOBP5 only binds β-ionone [33].

The wheat blossom midge *Sitodiplosis mosellana* Gehin (Diptera: Cecidomyiidae) is one of the most damaging pests of wheat, causing serious yield loss in some parts of the Northern hemisphere [34,35]. This midge oviposits on wheat spikes primarily before anthesis, but exhibits apparently different preference among wheat varieties [36,37]. Studies have showed that volatiles emitted from wheat spikes are crucial in midge oviposition site selection [38]. Antennae of the midge possess numerous sensillar hairs or pegs, where odorant reception occurs [39]. Currently, monitoring of adult *S. mosellana* populations mainly depends on net sweeping and sex pheromone traps [40,41]. However, net sweeping is time-consuming. Moreover, adults are small in size (3 mm in body length) and most active right after dusk, making their identification and quantification hard. Using a pheromone trap may not detect outbreaks in a timely manner because the pheromone (i.e., 2, 7-nonanediyl dibutyrate) affects only males, however, females are better fliers and greater in number than the males [42,43]. More importantly, the female midge is directly responsible for infestation by oviposition [36]. Therefore, development of new strategies based on olfactory cues from host plants could represent a significant improvement of current monitoring of *S. mosellana*.

We have previously characterized three of the 26 candidate OBPs currently annotated in *S. mosellana* [44]. We demonstrate that these three OBPs, SmosOBPs 11, 16, and 21, differ in their affinity to wheat volatiles [45]. To further explore OBP functionality in this midge, here we cloned another two *OBP* genes highly expressed in female antennae (i.e., *SmosOBP12* and *SmosOBP17*), and evaluated their selectivity and sensitivity to different host volatile components using a fluorescence competitive binding assay [46]. We also examined behavioral responses of adult *S. mosellana* to odorant molecules. Results have shed more light on the mechanism of host searching in *S. mosellana.*

## 2. Materials and Methods

### 2.1. Experimental Insects

*S. mosellana* cultures were established by taking soil samples from a wheat field with severe *S. mosellana* damage at Zhouzhi, Shaanxi Province, China, during February 2017, and the soil samples with insects were stored at 4 °C. When needed, samples were transferred to pots (12 cm in diameter) and incubated at 24 °C with 70% relative humidity (R.H.). Pots were periodically watered to maintain moisture for insect development [47]. Adults generally emerged within 3 weeks under these conditions. Females were used for antenna collection and behavioral assays, considering the fact that in the field, females are more abundant and possess stronger flight capacity, and are responsible for infestation due to their oviposition [42,43]. It should be noted that females used in the experiments may have mated since they came from containers with mixed sex.

### 2.2. RNA Isolation, cDNA Synthesis, and OBP Cloning

Female antennae (about 300) were carefully removed, immediately frozen in liquid nitrogen, and stored at −80 °C until use. Total RNA from dissected antennae was extracted with the RNAsimple Total RNA Kit (Tiangen, Beijing, China) according to the user’s manual. The integrity of total RNA was examined with 1% agarose gel electrophoresis, and the purity was determined by a spectrophotometer, i.e., the OD_260_/OD_280_ value. cDNAs were synthesized from 1.0 μg total RNA using PrimeScript™ II 1st Strand cDNA Synthesis kit (TaKaRa, Dalian, China) following the manufacturer’s instructions.

We selected *SmosOBP12* and *SmosOBP17* as target genes because of their specific expression and/or high abundance in *S. mosellana* female antennae [44]. Partial sequences of two candidate SmosOBPs were identified based on the previously annotated transcriptome of *S. mosellana*. Of these, the 3′ sequence was intact for *SmosOBP17* but missing for *SmosOBP12.* To obtain the complete open reading frames (ORF), gene-specific primers for 3′-RACE for *SmosOBP12* and 5′-RACE for both genes (Table 1) were synthesized based upon the identified unigene sequences. 5′-and 3′-RACE were performed with the 5′-Full RACE Kit with TAP and the 3′-Full RACE Core Set with PrimeScript™RTase (TaKaRa, Dalian, China) in accordance with the recommended protocols. The primary and nested PCR conditions were as follows: initial denaturation for 3 min at 94 °C; 20 cycles of 30 s at 94 °C, 30 s at 55 °C, and 60 s at 72 °C; a final extension of 10 min at 72 °C. The PCR products were purified using the DNA Purification kit (Tiangen, Beijing, China), ligated into the vector pMD-19T (TaKaRa, Dalian, China), and then transformed into *Escherichia coli* (*E. coli*) DH5α competent cells (Tiangen, Beijing, China), respectively. Three or more colonies were randomly selected for plasmid DNA extraction and sequencing (Invitrogen Biotechnology Co., Ltd., Shanghai, China).

The entire coding regions of *SmosOBP12* and *SmosOBP17* were finally PCR amplified with gene-specific primers (Table 1). For *SmosOBP17*, PCR reactions were carried out under the following conditions: 3 min at 95 °C; 30 cycles of 40 s at 95 °C, 50 s at 55 °C, 60 s at 72 °C; and 72 °C for 10 min. For *SmosOBP12*, a 35-cycle touchdown PCR was performed. The thermocycling program included denaturation at 95 °C for 3 min, 10 cycles of 40 s at 95 °C, 50 s at 60 °C, and 60 s at 72 °C with a 1 °C decrease of annealing temperature per cycle. The remaining 25 cycles consisted of 40 s at 95 °C, 50 s at 55 °C, 60 s at 72 °C; and a final extension of 72 °C for 10 min. Finally, PCR products were cloned and confirmed by sequencing analysis as described above.

### 2.3. Sequence and Phylogenetic Analyses

The online software ORF Finder (http://www.ncbi.nlm.nih.gov/gorf/gorf.html) was used to determine protein sequences of SmosOBP12 and SmosOBP17 from their cDNAs. The molecular weight and theoretical isoelectric points of these putative proteins were calculated with the Expasy server program (http://web.expasy.org/compute_pi/). N-terminal signal peptides were predicted using SignalP 5.0 server (http://www.cbs.dtu.dk/services/signalP-5.0). Sequence alignment of these two SmosOBPs with OBPs from other insects were carried out with the DANMAN 6.0 software (Lynnon Corporation, Pointe-Claire, QC, Canada). A phylogenetic tree was built by MEGA 10.0.5 (Temple University, Philadelphia, PA, USA) software using the neighbor-joining algorithm with 1000 bootstrap replications based on 41 OBP amino acid sequences from dipteran insects.

### 2.4. Prokaryotic Expression and Purification of SmosOBPs

Coding regions of *SmosOBP12* and *SmosOBP17* without signal peptides were PCR amplified and cloned into the pMD-19T vector (TaKaRa, Dalian, China). The constructs were then restricted by BamHI and HindIII (designed into the cloning primers, Table 1) and inserted in-frame into the expression vector pET28a (+) (Novagen, Madison, WI, USA) digested by the same restriction endonucleases. Sequence-confirmed pET28a/SmosOBP12 and pET28a/SmosOBP17 constructs were transformed into *E. coli* strains BL21 and Rosetta, respectively (Tiangen, Beijing, China).

Single colonies containing the verified insert sequences were cultured in Luria–Bertani (LB) media (containing 100 μg/mL kanamycin) in a shaker set at 220 rpm and 37 °C. The overnight culture was used to inoculate 500 mL fresh medium with kanamycin. Expression of recombinant SmosOBPs were induced by addition of isopropyl β-D-1-thiogalactoside (IPTG) to a final concentration of 0.5 mM when the culture reached OD_600_ of 0.6–0.8. After a 5 h induction, cells were harvested by centrifugation at 6000 g for 10 min. Cell pellets were homogenized in Tris-HCl buffer (20 mM, pH 7.4), lysed with 0.4 mg/mL lysozyme, sonicated on ice (10 s, 15 passes), and centrifuged (12,000 g for 30 min). Recombinant SmosOBPs were examined by SDS-PAGE. Expressed proteins in inclusion bodies were denatured with 8 M urea and renatured by extensive dialysis following the procedure of a previous study [48].

Solubilized SmosOBPs were purified using the Ni-NTA His·Bind Resin affinity column (7Sea Pharmatech Co., Shanghai, China) following the procedure described in Li et al. (2016) [46], examined on 15% SDS-PAGE, and further verified by Western blot analysis using the mouse anti-His tag monoclonal antibody (Sino Biological, Beijing, China). Protein concentrations were determined by the Bicinchoninic Acid (BCA) Protein Assay Kit (GeneStar, Beijing, China).

### 2.5. Fluorescence Competitive Binding Assays

To determine binding affinities of SmosOBP12 and SmosOBP17 to host plant volatile compounds, fluorescence competitive binding assays were conducted using 1-N-phenyl-naphthylamine (1-NPN) as the fluorescent probe. Twenty-eight volatile compounds from winter wheat (Table 2) were selected based on our previous research [49]. Both the probe and candidate ligands were dissolved in chromatographic-grade methanol to obtain 1 mM stock solutions, whereas recombinant SmosOBPs were diluted in 20 mM Tris-HCl (pH 7.4) to 2 μM. Fluorescence intensity was detected on an F-4600 fluorescence spectrophotometer (Hitachi, Tokyo, Japan) with a 1 cm light path quartz cuvette. Slits of both excitation and emissions were 10 nm in width. The probe was excited at 337 nm, and emission spectra were recorded between 370 and 550 nm.

To obtain the dissociation constant (K_d_) of SmosOBPs and 1-NPN as a measurement of their binding affinity, 1 mL of a 2 µM solution of each protein was titrated with aliquots of 1 mM 1-NPN to final concentrations of 0–24 µM, and the fluorescence intensities at the maximum fluorescence emission were recorded against the concentration of 1-NPN. Affinities of SmosOBPs to tested volatile ligands were measured by competition assays: to the 1 mL solution containing 2 μM recombinant protein and 2 μM 1-NPN, 1 mM solution of each putative volatile ligand (in a 2 µL aliquot) was added to final concentrations of 2–14 μM for SmosOBP12 and 2–40 μM for SmosOBP17, respectively. Maximal fluorescence intensities were plotted against ligand concentrations. Data were obtained from three independent measurements.

The K_d_ value was calculated using GraphPad Prism 5 (GraphPad Software Inc., La Jolla, CA, USA) via nonlinear regression for a unique site of binding. The inhibition constant (K_i_) of each ligand competitor was calculated from the corresponding IC_50_ value (the concentration of competitor displacing 50% of initial fluorescence intensity) according to the equation: K_i_ = [IC_50_]/(1 + [1-NPN]/K_1-NPN_), where [1-NPN] is the free concentration of 1-NPN, and K_1-NPN_ is the dissociation constant of the SmosOBPs/1-NPN complex [50]. We considered ligand binding affinity to SmosOBPs very strong (K_i_ ≤ 5 µM), strong (5 µM <K_i_ ≤ 15 µM), medium (15 µM <K_i_ ≤ 30 µM), and weak (K_i_ > 30 µM) in this study.

### 2.6. Y-Tube Olfactometer Bioassays

Behavioral responses of *S. mosellana* females to the 28 volatile ligands were measured in a glass Y-tube olfactometer. The base and two arms of the Y-tube are 15 cm in length and 25 mm in internal diameter. The angle between arms is 60°. A 10 μL aliquot of the test chemical in paraffin oil at a concentration of 20 μg/μL [45] was applied to a filter paper strip (20 × 20 mm), which was allowed to evaporate for 20 s and then placed into one of the two odor bottles. The other odor bottle contained a filter paper strip treated with 10 μL of paraffin oil as the control. Moist, activated-charcoal filtered air entered both odor bottles connected by Teflon tubing to their respective arms of the Y-tube. The airflow rate through the olfactometer was 100 mL/min, which was measured by a float rotor meter. A newly emerged female *S. mosellana* adult was introduced into the end of the base of the Y-tube, which was then immediately covered with a dark paper box. An office lamp (20 W) illuminated the joint of two arms to facilitate observation. The choice was made if the *S. mosellana* female walked or flew 5 cm past the Y junction within 5 min and remained there for at least 15 s. Otherwise, it would be recorded as no-choice. Odor source was renewed for every five individuals. After testing for 10 individuals, the Y-tube was thoroughly cleaned with 95% ethanol and dried, and the treatment and control arms were switched to avoid the directional bias. After one odor source was tested, the Y-tube, odor bottles, and Teflon tubing were cleaned and dried before reuse. All experiments were conducted in a laboratory with a temperature of 25 ± 1 °C from 5:00 to 9:00 p.m. Sixty female adults were tested in each treatment group, and each individual was used only once.

The choice response of insects in each treatment group was analyzed by the chi-square test using SPSS 20.0 software (Chicago, IL, USA). Non-selecting insects were recorded but not included in the statistical analysis.

## 3. Results

### 3.1. Characterization of SmosOBP cDNAs

**Figure 1:**
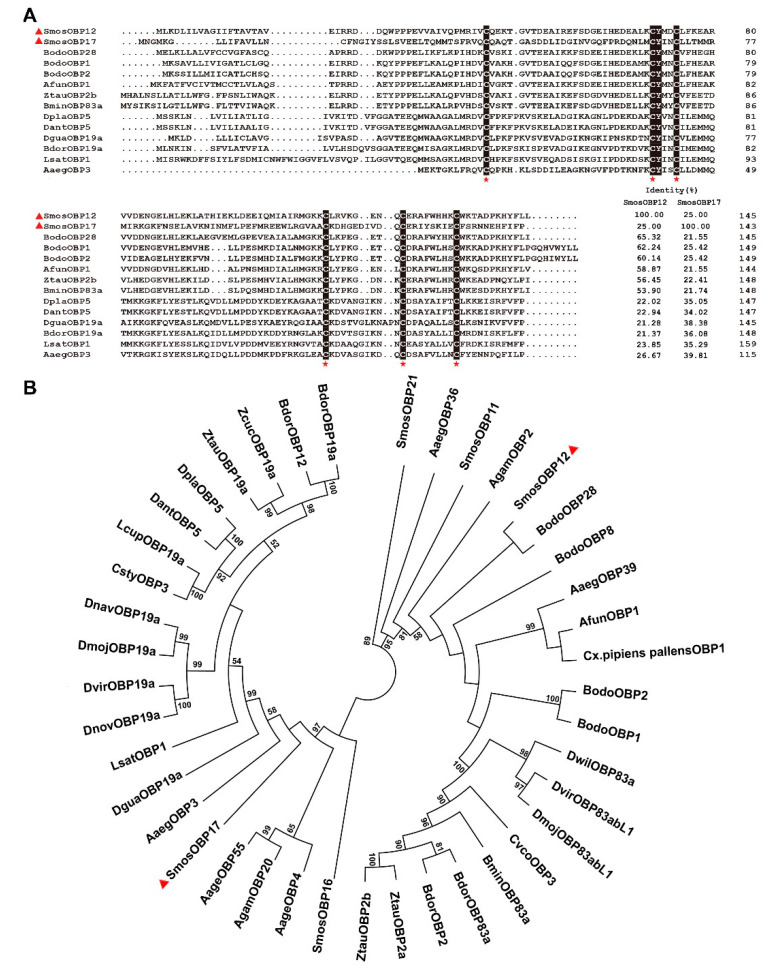
Multiple sequence alignment (**A**) and phylogenetic analysis (**B**) of *Sitodiplosis mosellana* odorant-binding proteins (SmosOBPs) and dipteran homologs. Six conserved cysteines are marked with red asterisks. SmosOBP12 and SmosOBP17 are indicated by red triangles. The phylogenetic tree was built using the neighbor-joining method with 1000 bootstrap replications and presented with a cutoff value of 50. Gene names and GenBank accession numbers of 41 OBPs are as follows: *S. mosellana* (SmosOBP12, MG585343; SmosOBP17, MG585345; SmosOBP11, MG585344; SmosOBP16, KF782364; SmosOBP21, KF782358); *Bradysia odoriphaga* (BodoOBP28, AWC08439.1; BodoOBP1, ANA52575.1; BodoOBP2, ANA52576.1; BodoOBP8, AWC08439.1); *Anopheles funestus* (AfunOBP1, ADQ01701.1); *Zeugodacus tau* (ZtauOBP2b, AKB92821.1; ZtauOBP19a, ALS40418.1; ZtauOBP2a, AKB92820.1); *Bactrocera minax* (BminOBP83a, AYN70647.1); *Delia platura* (DplaOBP5, BAS69445.1); *Delia antiqua* (DantOBP5, BAI82445.1); *Drosophila Guanche* (DguaOBP19a, SPP78474.1); *Bactrocera dorsalis* (BdorOBP19a, AKI28998.1; BdorOBP12, AKM45830.1; BdorOBP2, AGO28153.1; BdorOBP83a, XP_011212472.1); *Liriomyza sativae* (LsatOBP1, ALZ41694.1); *Aedes aegypti* (AaegOBP3, AAEL000051; AaegOBP4, AAEL000073; AaegOBP36, AAEL008011; AaegOBP39, AAEL009449; AaegOBP55, AAEL012377); *Anopheles gambiae* (AgamOBP2, AAO12083.1; AgamOBP20, AAO12087.1); *Drosophila navojoa* (DnavOBP19a, XP_017965215.1); *Drosophila mojavensis* (DmojOBP19a, XP_002011011.1; DmojOBP83abL1, XP_001999215.1); *Drosophila virilis* (DvirOBP19a, XP_002058161.1; DvirOBP83abL1, XP_002058580.1); *Drosophila novamexicana* (DnovOBP19a, XP_030568576.1); *Zeugodacus cucurbitae* (ZcucOBP19a, XP_011187213.1); *Lucilia cuprina* (LcupOBP19a, XP_023294703.1); *Calliphora stygia* (CstyOBP3, AID61296.1); *Culex pipiens pallens* (Cx.pipiens pallensOBP1, AMQ13063.1); *Drosophila willistoni* (DwilOBP83a, XP_002073644.2); *Carpomya vesuviana* (CvesOBP3, AMY98994.1).

The full-length cDNAs of *SmosOBP12* (accession No. MG585343) and *SmosOBP17* (MG585345) were obtained by RACE-PCR and ordinary PCR using gene-specific primers. ORFs of *SmosOBP12* and *SmosOBP17* encoded for proteins of 145 and 143 amino acid residues (Figure S1), respectively. The predicted molecular weight and isoelectric point for SmosOBP12 were 14.97 kDa and 5.38, and 14.08 kDa and 5.14 for SmosOBP17. Sequence analysis indicated that both SmosOBPs possessed signal peptides of 18–22 amino acid residues at their N-termini (Figure S1). Moreover, they had the typical signature of six cysteines in the pattern of C_1_-X_26_-C_2_-X_3_- C_3_-X_40_-C_4_-X_8–10_-C_5_-X_8_-C_6_ (Figure 1 and Figure S1). Therefore, they belong to the classic OBP subfamily [51].

Amino acid sequence alignment of SmosOBPs with homologues from other dipterans indicated that SmosOBP12 shared the highest sequence identities (65.3%) to *Bradysia odoriphaga* OBP28 (BodoOBP28) followed by BodoOBP1 (62.2% identity); SmosOBP17 had the highest sequence identities (39.8%) to *Aedes aegypt* OBP3 (AaegOBP3) and *Drosophila guanche* OBP19a (DguaOBP19a) (38.4% identity). The two SmosOBPs displayed a relatively lower homology (25.0%) (Figure 1A). The phylogenetic analysis grouped the 41 OBPs from *S. mosellana* and other dipterans into two branches, and SmosOBP12 and SmosOBP17 fell into different branches. The closest homologues were BodoOBP28 for SmosOBP12, and AaegOBP3 for SmosOBP17 (Figure 1B).

### 3.2. Expression and Purification of SmosOBPs

**Figure 2:**
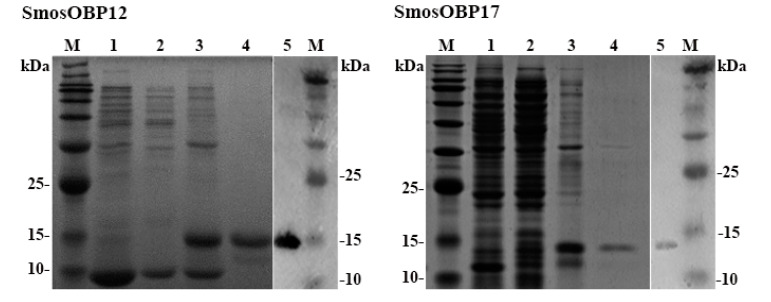
Bacterially expressed SmosOBP12 and SmosOBP17. SDS-PAGE of un-induced recombinant *Escherichia coli* harboring pET28a (+)/SmosOBPs (lane 1), supernatant (lane 2) and precipitate (lane 3) of IPTG-induced *E. coli*, and Ni-NTA affinity-purified SmosOBPs (lane 4). Western blot analysis of purified SmosOBPs (lane 5). M, molecular weight markers.

Both SmosOBPs were successfully expressed in the prokaryotic expression system after IPTG induction but were present in inclusion bodies (Figure 2). Yields of the renatured proteins were 0.89 mg/mL for SmosOBP12 and 0.60 mg/mL for SmosOBP17. Specific bands of expected sizes corresponding to the purified SmosOBP12 and SmosOBP17 were detected on both SDS-PAGE and Western blot analysis (Figure 2).

### 3.3. Distinct Binding Affinities of SmosOBPs12 and 17 to Host Plant Volatiles

**Figure 3:**
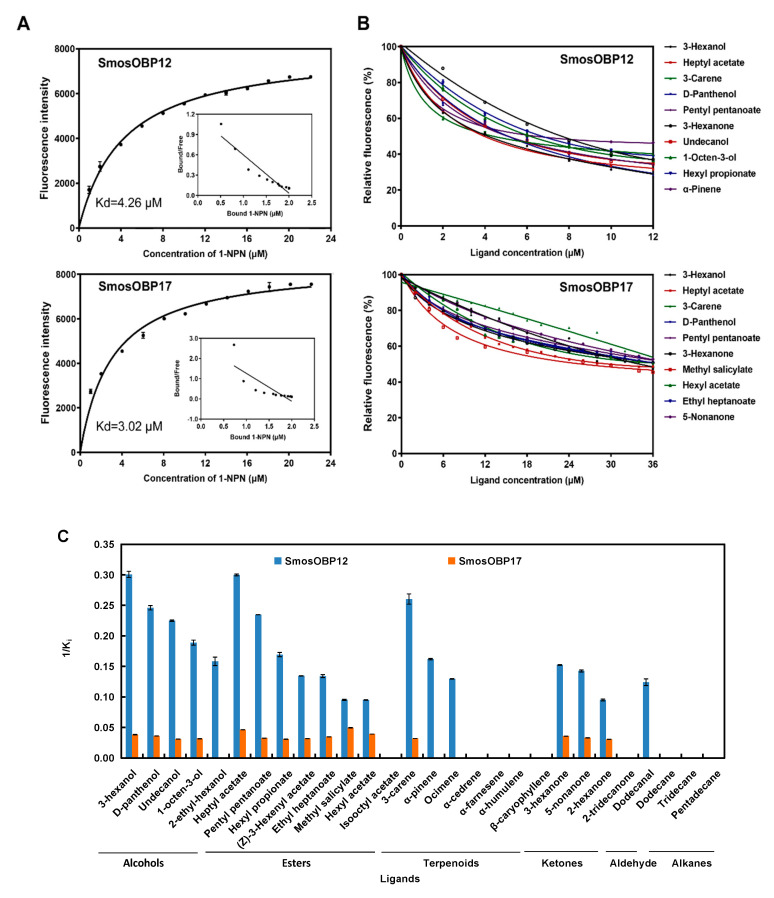
Ligand-binding assays of recombinant SmosOBP12 and SmosOBP17. (**A**) Binding curves and Scatchard plots of SmosOBP/1-NPN associations. (**B**) Fluorescence competitive binding curves of SmosOBPs to the 10 tightest bound ligands. (**C**) Affinities (1/K_i_) of tested ligands to the two SmosOBPs. Data are means of three independent experiments. The error bars of some points may not be clearly seen because they are too small to be displayed.

To explore the function of the two SmosOBPs in perception of wheat plant volatiles, we first measured their binding affinities to the fluorescent probe 1-NPN. Based on the changes in the fluorescence intensity, dissociation constants (K_d_) with 1-NPN were 4.26 and 3.02 μM for SmosOBP12 and SmosOBP17, respectively (Figure 3A).

Recombinant SmosOBP12 and SmosOBP17 exhibited distinct binding to tested compounds (Figure 3B,C). SmosOBP12 bound to 19 odorants, while SmosOBP17 bound to 15 odorants (Figure 3C, Table 2). For those commonly shared volatile substrates, SmosOBP12 showed stronger binding than SmosOBP17. Specifically, SmosOBP12 showed very strong (K_i_ < 5 μM) or strong affinities (K_i_ = 10.25–10.42 μM) to all five tested alcohols, three of the seven terpenoids (ocimene, α-pinene, and 3-carene), seven of the eight esters (except isooctyl acetate), three of the four ketones (3-hexanone, 2-hexanone, and 5-nonanone), and dodecanal. In contrast, SmosOBP17 did not bind to 2-ethylhexanol, ocimene, α-pinene, and dodecanal; and showed medium (K_i_ = 20.13–28.76 μM) or weak affinities (K_i_ = 30.19–32.61 μM) to the other four alcohols, seven esters, three ketones, and 3-carene. Interestingly, two SmosOBPs displayed obviously different binding preference for several compounds with the same molecular formula, such as C_10_H_16_ (i.e., 3-carene, α-pinene and ocimene), C_9_H_18_O_2_ (i.e., heptyl acetate, ethyl heptanoate, and hexyl propionate), C_10_H_20_O_2_ (i.e., pentyl pentanoate and isooctyl acetate) and C_6_H_12_O (i.e., 2-hexanone and 3-hexanone). They had stronger affinities with 3-carene, heptyl acetate, pentyl pentanoate, and 3-hexanone compared to their isomers. Clearly, the best four ligands were 3-hexanol, heptyl acetate, 3-carene, and D-panthenol for SmosOBP12 with K_i_ value ranging from 3.33 to 4.07 μM; and methyl salicylate, heptyl acetate, hexyl acetate, and 3-hexanol for SmosOBP17 with K_i_ value ranging from 20.13 to 26.05 μM (Figure 3B, Table 2). On the other hand, all three tested alkanes, four terpenoids with the same molecular formula of C_15_H_24_ (i.e., α-cedrene, α-farnesene, α-humulene, and caryophyllene), isooctyl acetate, or 2-tridecanone did not show any affinity to either of the SmosOBPs.

### 3.4. Behavior of S. mosellana in Y-Tube Olfactometer Assays

**Figure 4:**
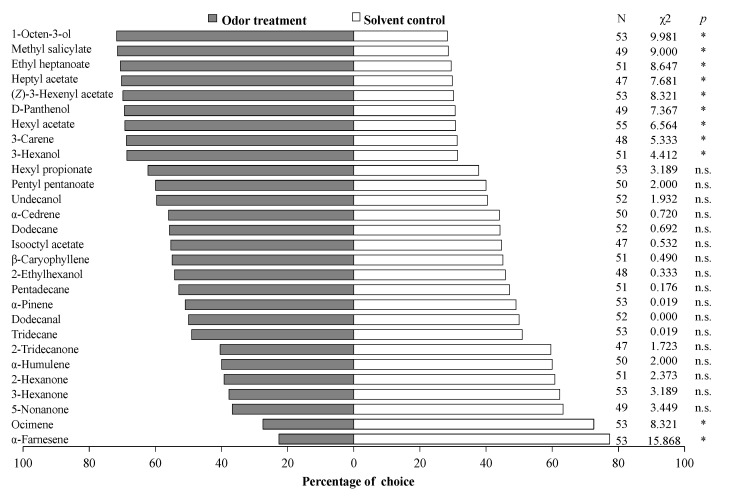
Responses of female *Sitodiplosis mosellana* adults to synthetic odors derived from wheat volatiles. All tested compounds subjected to Y-tube olfactometer assays were diluted with liquid paraffin to a final concentration of 20 μg/μL and liquid paraffin was used as the solvent control. N marks the number of individuals that made a choice out of 60 tested insects, and the insects that did not make a choice are excluded from the statistical analysis. Asterisk denotes significant difference (*p* *<* 0.05), and n.s. indicates no significant difference by chi-square tests.

Among the 28 volatiles tested, 11 could elicit obvious behavioral response of *S. mosellana* female adults. Adults showed a significant attraction to nine of them, including 1-octen-3-ol (χ^2^ = 9.981, *p* = 0.002), methyl salicylate (χ^2^ = 9.000, *p* = 0.003), ethyl heptanoate (χ^2^ = 8.647, *p* = 0.003), heptyl acetate (χ^2^ = 7.681, *p* = 0.006), (*Z*)-3-hexenylacetate (χ^2^ = 8.321, *p* = 0.004), D-panthenol (χ^2^ = 7.367, *p* = 0.007), hexyl acetate (χ^2^ = 6.654 *p* = 0.010), 3-carene (χ^2^ = 5.333, *p* = 0.021), and 3-hexanol (χ^2^ = 4.412, *p* = 0.036). In contrast, they also displayed a significant repulsion to α-farnesene (χ^2^ = 15.868, *p* = 0.000) and ocimene (χ^2^ = 8.321, *p* = 0.004). The remaining 17 compounds were neither attractive nor repulsive to *S. mosellana* (Figure 4).

## 4. Discussion

Annotation of insect genomes and antennal transcriptomes has resulted in identification of numerous *OBP* genes. It is generally believed that those abundant in adult antennae are essential for olfaction, enabling insects to detect volatile compounds for mating, foraging, and locating suitable oviposition sites [28,46,52]. Of the two *S. mosellana OBP* genes cloned in this study, *SmosOBP12* is highly and specifically expressed in antennae of female adults, and *SmosOBP17* is mainly expressed in antennae of both sexes [44], implying potential roles in the detection of host plant volatiles. Limited sequence identity between the two proteins (25%) (Figure 1) suggests their functional divergence.

*S. mosellana* thrives mainly on wheat, and depends heavily on volatile cues of wheat ears before anthesis to select wheat varieties for oviposition [38,49]. To elucidate differential roles played by SmosOBPs in perceiving host odors, volatiles from wheat ears were used as putative ligands in fluorescence competitive binding assays in the present study. Similar to many other insect OBPs such as OBPs 3 and 8 in *Agrilus mali* [19], OBPs 2 and 6 in *Chrysoperla sinica* [31], OBPs 1 and 2 in *Chilo suppressalis* [53], and OBP1 in *Adelphocoris lineolatus* [16], SmosOBPs 12 and 17 could selectively recognize functional groups of host odorants. SmosOBPs 12 and 17 failed to bind any of the alkanes tested, but could effectively bind to most alcohols, esters, and ketones, as well as terpenoid compounds, and SmosOBP12 also bound dodecanal (Table 2). Furthermore, we found that the carbon chain length and steric configuration of odorant molecules also affected their interaction with SmosOBPs. For example, the two SmosOBPs could bind ketones and terpenoids that have short carbon chains (C6–C10), but not those with long chains (C13–C15) (Table 2). They showed greater affinities for 3-carene, ethyl heptanoate, and 3-hexanone than their isomers α-pinene, isooctyl acetate, and 2-hexanone, respectively. Consistently, *Spodoptera litura* GOBP1 and *Loxostege sticticalis* GOBP2 prefer shorter-chain esters or aldehydes to longer-chain forms [27,30]. Likewise, *G. molesta* OBP11 and *A. lineolatus* OBP1 can distinguish isomers of ketones or terpenoids [16,46].

Notably, SmosOBP12 displayed a broader ligand-binding spectrum with higher affinity to alcohols, esters, ketones, terpenoids, and aldehydes (K_i_ < 10.5 μM) compared to SmosOBP17 (K_i_ > 20.1 μM) as well as to SmosOBPs 11, 16, and 21, three antenna-specific OBPs that we previously reported [45], indicating that SmosOBP12 may play more crucial roles than other SmosOBPs in perceiving host plant volatiles. Similar findings were also reported before [30,52,54]. For instance, GOBP2 of *Agrotis ipsilon* binds a wider range of plant odorants with a greater affinity than AipsGOBP1 [28]. OBP4 from *M. mediator* has a broader binding spectrum as well as stronger affinity than MmedOBPs 5 and 7 with the tested host volatiles [33].

We have shown earlier that 3-hexanol, 1-octen-3-ol, D-panthenol, (*Z*)-3-hexenylacetate, hexyl acetate, methyl salicylate, heptyl acetate, and ethyl heptanoate elicit strong electrophysiological responses on the female antennae of *S. mosellana* (Cheng et al., 2020). In our behavioral tests here, these compounds significantly attracted *S. mosellana* females (Figure 4). It is thus conceivable that some of these volatiles could be used for development of *S. mosellana* attractants for monitoring and management of this pest. SmosOBP12 most likely facilitates transportation of these compounds to olfactory receptors due to its particularly strong affinity to the volatiles (K_i_ < 10.5 μM) (Table 2). In contrast, ocimene exerted a strong repelling effect on female *S. mosellana* (Figure 4), which may explain its high abundance in less-preferred wheat varieties Shanmai 139 and Jinmai 47 for *S. mosellana* oviposition [49]. SmosOBPs 11, 16, 17, and 21 did not bind this compound, but SmosOBP12 exhibited high affinity to it (K_i_ = 8.2 μM), implying that ocimene is more likely to function in host selection through specific interaction with SmosOBP12. Notably, α-farnesene also repelled *S. mosellana* but showed no affinity to any of the five SmosOBPs identified up to date [45]. Presumably, other OBPs in *S. mosellana* are responsible for binding and transportation of this key odorant [44], and further study is necessary to fully elucidate molecular mechanisms underlying host selection in *S. mosellana*.

## 5. Conclusions

In conclusion, our in vitro binding and behavioral assays strongly suggested that both SmosOBP12 and SmosOBP17 could selectively detect and recognize host wheat volatiles which impact host selection behavior of *S. mosellana*. Combined with our earlier study on SmosOBPs11, 16, and 21, we concluded that SmosOBP12 may play the most prominent role in this process. Although application of RNA interference to confirm biological functions of *SmosOBPs* is the apparent follow-up study, it is not yet technically feasible at the present time for this particular species. Targeted technical development will no doubt be crucial in facilitating in vivo functional dissection of *SmosOBPs* and for OBP-based behavioral interference for monitoring and control of this key pest.

## Figures and Tables

**Table 1:** Primers used in cloning and expression of *Sitodiplosis mosellana* odorant-binding proteins 12 and 17 (SmosOBP12 and SmosOBP17).

Primer Name	Sequence (5′-3′)	Purpose
OBP12-outer	TACTCGTAAGCACTTCTTGCC	5′ RACE
OBP12-inner	CACTTCTTGCCCATGCGA	
OBP17-outer	GAAGAGCTAACGCAAATGATGAC	
OBP17-inner	CCGGAGCTTCTGATGATCTTATT	
OBP12-outer	GTCACCGACGAGGCGAT	3′ RACE
OBP12-inner	AGGCGATCCGAGAATTTAGTG	
OBP12-forward	TCATCAAGCCCAACTTCTGT	ORF cloning
OBP12-reverse	TTAAAGCAAGAAGTAATGTTTTGG	
OBP17-forward	CTATGGAAATATGAAATGTTC	
OBP17-reverse	ATGAACGGAATGAAAGGTTTACTGA	
OBP12-forward	CGGGATCCGTTGAAATACGTCGAGATGATC (BamHI)	*Escherichia coli* expression
OBP12-reverse	CCCAAGCTTTTAAAGCAAGAAGTAATGTTTTGGA (HindIII)	
OBP17-forward	CCCAAGCTTCTATGGAAATATGAAATGTTC (HindIII)	
OBP17-reverse	CGGGATCCAGTTTATCTGTTGAAGAGC (BamHI)	

Restriction endonucleases are shown in parentheses after primers, and restriction sites are underlined.

**Table 2:** Binding affinities of two SmosOBPs to wheat volatile compounds in fluorescence binding assays.

Compounds	CAS No.	Molecular Weight	Formula	SmosOBP12		SmosOBP17	
				IC_50_ (μM)	K_i_ (μM)	IC_50_ (μM)	K_i_ (μM)
Alcohols							
3-Hexanol	623-37-0	102.18	C_6_H_14_O	4.12 ± 0.07	3.33 ± 0.05	34.68 ± 0.34	26.05 ± 0.25
1-Octen-3-ol	3391-86-4	128.21	C_8_H_16_O	6.55 ± 0.12	5.29 ± 0.11	42.43 ± 0.09	31.88 ± 0.07
2-Ethyl hexanol	104-76-7	130.23	C_8_H_18_O	7.86 ± 0.31	6.36 ± 0.25	-	-
D-Panthenol	81-13-0	205.25	C_9_H_19_NO_4_	5.04 ± 0.08	4.07 ± 0.05	38.01 ± 0.20	28.55 ± 0.15
Undecanol	112-42-5	172.31	C_11_H_24_O	5.43 ± 0.03	4.39 ± 0.02	42.81 ± 0.14	32.16 ± 0.08
Terpenoids							
3-Carene	13466-78-9	136.23	C_10_H_16_	4.76 ± 0.15	3.84 ± 0.12	41.66 ± 0.05	31.29 ± 0.04
α-Pinene	80-56-8	136.23	C_10_H_16_	7.63 ± 0.06	6.17 ± 0.04	-	-
Ocimene	13877-91-3	136.23	C_10_H_16_	10.17 ± 0.04	8.24 ± 0.03	-	-
α-Cedrene	469-61-4	204.35	C_15_H_24_	-	-	-	-
α-Farnesene	502-61-4	204.35	C_15_H_24_	-	-	-	-
α-Humulene	6753-98-6	204.35	C_15_H_24_	-	-	-	-
β-Caryophyllene	87-44-5	204.35	C_15_H_24_	-	-	-	-
Esters							
(*Z*)-3-Hexenyl acetate	3681-71-8	142.20	C_8_H_14_O_2_	8.88 ± 0.02	7.18 ± 0.01	41.75 ± 0.19	31.36 ± 0.14
Hexyl acetate	142-92-7	144.21	C_8_H_16_O_2_	12.88 ± 0.09	10.42 ± 0.08	33.96 ± 0.03	25.51 ± 0.02
Methyl salicylate	119-36-8	152.15	C_8_H_8_O_3_	11.88 ± 0.24	9.62 ± 0.33	26.79 ± 0.27	20.13 ± 0.20
Heptyl acetate	112-06-1	158.24	C_9_H_18_O_2_	4.11 ± 0.02	3.34 ± 0.02	28.79 ± 0.09	21.63 ± 0.06
Ethyl heptanoate	106-30-9	158.24	C_9_H_18_O_2_	9.00 ± 0.11	7.29 ± 0.09	38.28 ± 0.12	28.76 ± 0.09
Hexyl propionate	2445-76-3	158.24	C_9_H_18_O_2_	7.28 ± 0.16	5.90 ± 0.12	43.08 ± 0.33	32.36 ± 0.24
Pentyl pentanoate	2173-56-0	172.27	C_10_H_20_O_2_	5.23 ± 0.01	4.24 ± 0.03	40.78 ± 0.08	30.64 ± 0.06
Isooctyl acetate	31565-19-2	172.27	C_10_H_20_O_2_	-	-	-	-
Ketones							
3-Hexanone	589-38-8	100.16	C_6_H_12_O	7.74 ± 0.04	6.26 ± 0.03	37.10 ± 0.06	27.87 ± 0.04
2-Hexanone	591-78-6	100.16	C_6_H_12_O	12.66 ± 0.08	10.25 ± 0.06	43.41 ± 0.04	32.61 ± 0.03
5-Nonanone	502-56-7	142.24	C_9_H_18_O	8.32 ± 0.14	6.72 ± 0.10	40.19 ± 0.37	30.19 ± 0.27
2-Tridecanone	593-08-8	198.35	C_13_H_26_O	-	-	-	-
Aldehyde							
Dodecanal	112-54-9	184.32	C_12_H_24_O	9.94 ± 0.44	8.06 ± 0.37	-	-
Alkanes							
Dodecane	112-40-3	170.34	C_12_H_26_	-	-	-	-
Tridecane	629-50-5	184.36	C_13_H_28_	-	-	-	-
Pentadecane	629-62-9	212.42	C_15_H_32_	-	-	-	-

‘-’, no detectable affinity.

## Supplementary Materials

The following are available online at https://www.mdpi.com/2075-4450/11/12/891/s1, Figure S1: Nucleotide and deduced amino acid sequences of SmosOBP12 and SmosOBP17 in Sitodiplosis mosellana. Start and stop codons are boxed. Predicated signal peptides are underlined. The six conserved cysteines are circled.
