# Supplementary material for: Functional Analysis of Odorant-Binding Proteins 12 and 17 from Wheat Blossom Midge Sitodiplosis mosellana Géhin (Diptera: Cecidomyiidae)
Source: Insects. 2020 Dec 17;11(12):891. doi: 10.3390/insects11120891 (PMC7767053; doi:10.3390/insects11120891)
Supplement: Supplementary file 1 [file insects-11-00891-s001.pdf]

SmosOBP12

```
1     ATGCTAAAAGATTTAATTTTAGTCGCTGGTATTATTTTCACCGCTGTTACAGCCGTTGAAATACGTCGAGATGATCAGTGGCCACCACCCGAAGTCGTTGCTATTGTTCAGCCAATGAGA
1      M  L  K  D  L  I  L  V  A  G  I  I  F  T  A  V  T  A  V  E  I  R  R  D  D  Q  W  P  P  P  E  V  V  A  I  V  Q  P  M  R
121   ATTGTGTGCCAAGAAAAAACTGGCGTCACCGACGAGGCGATCCGAGAATTTAGTGATGGTGAAATTCATGAAGACGAAGCGTTGAAATGCTACATGGATTGTCTATTTAAAGAAGCTCGG
41     I  V  C  Q  E  K  T  G  V  T  D  E  A  I  R  E  F  S  D  G  E  I  H  E  D  E  A  L  K  C  Y  M  D  C  L  F  K  E  A  R
241   GTTGTGGATGAAAATGGTGAATTACATTTGGAAAAACTTGCCACACACATCGAAAAATTGGACGAGGAAATTCAAATGATCGCCATTCGCATGGGCAAGAAGTGCTTACGAGTAAAAGGG
81     V  V  D  E  N  G  E  L  H  L  E  K  L  A  T  H  I  E  K  L  D  E  E  I  Q  M  I  A  I  R  M  G  K  K  C  L  R  V  K  G
361   GAAAATCAGTGTGAACGAGCTTTTTGGTATCATAAATGCTGGAAAACAGCCGATCCAAAACATTACTTCTTGCTTTAA
121    E  N  Q  C  E  R  A  F  W  Y  H  K  C  W  K  T  A  D  P  K  H  Y  F  L  L  *
```

SmosOBP17

```
1     ATGAACGGAATGAAAGGTTTACTGATTTTTGCAGTGCTTTTGAACTGCTTCAATGGCATCTATTCAAGTTTATCTGTTGAAGAGCTAACGCAAATGATGACGTCTTTTCGAGTGCAATGT
1      M  N  G  M  K  G  L  L  I  F  A  V  L  L  N  C  F  N  G  I  Y  S  S  L  S  V  E  E  L  T  Q  M  M  T  S  F  R  V  Q  C
121   CAAGCCCAAACCGGAGCTTCTGATGATCTTATTGATGGAATTAATGTGGGCCAATTTCCAAGAGACCAAAATTTAATGTGCTACATAAATTGTTTACTTACAATGATGCGAATGATAAGG
41     Q  A  Q  T  G  A  S  D  D  L  I  D  G  I  N  V  G  Q  F  P  R  D  Q  N  L  M  C  Y  I  N  C  L  L  T  M  M  R  M  I  R
241   AAGGGAAAATTCAATTCTGAATTGGCAGTTAAGAATATAAATATGTTCTTGCCTGAATTTATGCGCGAAGAATGGTTGAGGGGAGTTGCAGCATGCAAAGATCATGGAGAAGATATTGTC
81     K  G  K  F  N  S  E  L  A  V  K  N  I  N  M  F  L  P  E  F  M  R  E  E  W  L  R  G  V  A  A  C  K  D  H  G  E  D  I  V
361   GATCAATGCGAAAGGATATATTCAAAGATTGAATGTTTCTCCAGAAATAACGAACATTTCATATTTCCATAG
121    D  Q  C  E  R  I  Y  S  K  I  E  C  F  S  R  N  N  E  H  F  I  F  P  *
```

Figure S1. Nucleotide and deduced amino acid sequences of *SmosOBP12* and *SmosOBP17* in *Sitodiplosis mosellana.* Start and stop codons are boxed. Predicated signal peptides are underlined. The six conserved cysteines are circled.
